# Chloride Homeostasis Failure in Human Disease: KCC2/NKCC1 Microdomain Dysfunction as a Driver of Cortical Network Collapse

**DOI:** 10.3390/ijms27073184

**Published:** 2026-03-31

**Authors:** Dan Dumitrescu, Stefan Oprea, Raluca Tulin, Adrian Vasile Dumitru, Octavian Munteanu, George Pariza

**Affiliations:** 1 Faculty of General Medicine, “Carol Davila” University of Medicine and Pharmacy, 050474 Bucharest, Romania; dan.dumitrescu@umfcd.ro (D.D.);; 2 Department of General Surgery, “Carol Davila” University of Medicine and Pharmacy, 050474 Bucharest, Romania; 3 Department of Anatomy, “Carol Davila” University of Medicine and Pharmacy, 050474 Bucharest, Romania; 4 Department of Pathology, Faculty of Medicine, “Carol Davila” University of Medicine and Pharmacy, 030167 Bucharest, Romania; 5 Puls Med Association, 051885 Bucharest, Romania

**Keywords:** chloride microdomains, kcc2 regulation, nkcc1 reactivation, gabaergic polarity, astrocytic buffering, inhibitory instability, cortical hyperexcitability

## Abstract

The regulation of chloride levels is a crucial part of controlling inhibitory signals, but does not occur uniformly throughout the body. Recent data suggest that chloride is regulated within localized “microdomains” which are defined by the interaction of KCC2 and NKCC1, structural restraints on cells due to their internal structure, the metabolic condition of the cell, and the external environment modified by astrocytes. The gradients of chloride concentrations within these compartment-specific microdomains define the local chloride reversal potential, and thereby determine the directionality (i.e., whether excitatory or inhibitory), magnitude, and timing of GABAergic inhibition. The disruption of this organized chloride gradient within microdomains impairs the stability of inhibitory activity at multiple levels of integration, including dendritic input, spike timing, interneuron synchronization, and network oscillation. Disturbances in inhibitory stability have been found in a variety of diseases, including epilepsy, neonatal seizure, neuropathic pain, and schizophrenia-spectrum disorders. This supports the hypothesis that disturbances in chloride homeostasis lead to a loss of stability in cortical circuits. This review will provide a synthesis of the molecular, spatial, and circuit level principles involved in the regulation of chloride and discuss how failures of these mechanisms produce clinically relevant disturbances in inhibitory signal processing. In addition, we will be discussing new therapeutic strategies for the restoration of chloride homeostasis, including KCC2 repair, selective modulation of NKCC1, targeting astrocytes, and microenvironmental engineering. Overall, the studies reviewed here provide a unified model for understanding the pathophysiology of inhibitory dysfunction, and demonstrate that the regulation of chloride microdomains provides a novel and promising area of research for translational intervention.

## 1. The Chloride Axis as a Dynamic Computational Variable in Human Cortex

Chloride homeostasis was classically thought to be a uniform property of neurons, characterized by a constant and stable intracellular chloride concentration (Chloride), a fixed reversal potential (ECl) and GABA(A) receptor-induced inhibitory action [1]. Recent studies using highly resolved imaging and electrophysiology have shown that the intracellular chloride concentration is spatially and temporally heterogeneous. In addition, cortical neurons contain sub-cellular “chloride micro-domains” with varying local concentrations of chloride, generating compartment specific ECl values that modulate the efficacy and polarity of inhibition in parallel with the neuronal synaptic computation [2]. These chloride micro-domains develop due to the interaction of several factors including, but not limited to; transporter distribution, impermeable anions and gradients, cell shape/geometry, astrocyte buffering of chloride, position of mitochondria, structure of cytoskeleton, and synaptic activation. These factors act at sub-micron spatial scales and develop on time-scales similar to those of the neuronal spike [3].

The intracellular distribution of chloride is limited by cytoplasmic tortuosity, interactions with fixed anionic macromolecules, and geometric compartmentalization of dendritic, spine and axonal segments. These constraints create spatially heterogenous electrostatic environments that can effect chloride distribution independent of the activity of transporters. Local changes in the concentration of impermeable anions can also produce localized changes in the ECl value that can result in changes to the functional efficacy of inhibitory synaptic input during active periods [4,5].

Transporter localization also serves to refine these gradients. KCC2 is located in compartment specific nanodomains that determine the local extrusion capacity while NKCC1 is involved in transporting chloride into certain neuronal and glial compartments. The ratio of these two opposing fluxes will determine the local chloride dynamics in each micro-domain and establish local inhibitory polarity [6,7].

Under active synaptic conditions, chloride dynamics can limit the ability of inhibitory function. The influx of chloride associated with inhibitory synaptic activation must be quickly counteracted by transport and glial buffering. During periods of excessive synaptic activity, the buffering capacity of glia and transport can exceed its capabilities resulting in a local increase in chloride concentration that can cause a change in the ECl value and therefore reduce or reverse inhibitory efficacy. These effects are especially pronounced in areas where diffusion is limited by geometric compartmentalization [8,9].

Astrocytes also serve to regulate the extracellular ionic environment through their coordinated regulation of water flow, potassium levels, and volume dynamics. Astrocyte morphological changes can alter perisynaptic geometry and influence chloride redistributions to create a coupled neuron-glia system. Therefore, inhibitory function is determined by both intracellular transport and extracellular boundary conditions [10,11].

On a circuit level, chloride gradients play a significant role in determining inhibitory precision. Different interneuronal populations place different demands on chloride regulation based on the specific compartment in which they reside. Small changes in ECl can therefore significantly alter the amplitude, timing, and phase relationships of inhibitory synaptic inputs. These changes can significantly alter oscillatory coherence, synaptic integration, and gain control throughout entire cortical networks [12,13].

Altered chloride homeostasis disrupts inhibitory control over time. Altered chloride homeostasis is correlated with both desynchronized network activity and hyper-synchronized transitions indicative of pathological states. Moreover, the correlation between altered chloride homeostasis and pathological states is much stronger than the correlation between disrupted synaptic strength and pathological states [14,15].

The overall effect of these studies was to establish a conceptual structure that could be used to view chloride gradient dynamics as regulatory elements of inhibitory synaptic polarity and network stability. The conceptual structure described above provides a basis for the integration of the molecular, spatial, and circuit-level mechanisms by which chloride levels are regulated, with an emphasis on KCC2 and NKCC1 function; neuron-glial interaction; and the influence of microdomain dynamics on both physiological and pathological states. While there may still be some areas where this discipline will grow, the existing body of evidence appears to be sufficient to create a coherent multi-scale framework for understanding dysfunction dependent upon chloride levels. The current understanding of chloride regulation has been derived from a wide variety of complimentary experimental systems. While it remains technically challenging to directly characterize chloride microdomains in the human brain, the convergence of molecular, cellular and electrophysiological data support the concepts described in this paper. In this sense, the limitations to the development of this model can be seen less as limitations to the model per se, but more as an expression of the complexity of investigating chloride regulation at the highest level of spatial and temporal resolution. As such, future studies using high resolution and translational approaches should focus on improving (refining) this emerging model rather than completely overhauling it.

## 2. KCC2 Extrusion Mechanisms: Molecular, Spatial, and Activity-Dependent Regulation

### 2.1. Multi-Layered Phospho-Switch Logic and Conformational Landscapes

Phosphorylation of the C-terminal region of KCC2 allows for conformational changes (switches) which provide fine tuning of chloride extrusion. These switches are organized in a hierarchical fashion. Phosphorylation of each switch affects the availability and influence of other switches. This creates a phosphoregulatory landscape that produces a wide range of functional states of KCC2 based on the number and arrangement of phosphate groups attached to these sites [16,17]. The landscape of these switches has been shown to be responsive to a variety of stimuli including intracellular chloride levels, synaptic activity, metabolic status, and trophic signaling. The WNK-SPAK signaling pathway acts as a major coupling mechanism between intracellular chloride levels and KCC2 conformation. This pathway regulates transitions between the various functional states of the transporter [18]. Other signaling pathways also influence KCC2’s ability to couple ions and regulate the structural arrangement of the transporter. For example, glutamate receptor activation can affect the ability of the transporter to bind ions [19]. All of these influences occur through allosteric interactions between the different regions of the protein. Therefore, regulation of KCC2 occurs through a distributed mechanism rather than through separate modules [20].

Recent developments in methodology have supported this dynamic model. Studies using cryo-electron microscopy have identified multiple functional states of KCC2. Additionally, studies using phosphospecific imaging have demonstrated that KCC2 undergoes rapid, activity dependent changes in phosphorylation. These changes in phosphorylation lead to rapid changes in the functional states of the transporter [21]. Furthermore, live-cell imaging has shown that KCC2 dynamically adjusts its chloride extruding capability in response to synaptic activity. This allows for local stabilization of chloride gradients in response to changing demands [22].

### 2.2. Nanodomain Specialization, Spatial Logic, and Mesoscale Microarchitecture

Functionality of KCC2 is influenced by its organization into membrane nanodomains. Nanodomains vary in their densities and dynamics among neuronal compartments. They reflect both local synaptic environments and ionic environments [23,24]. The high density of KCC2 in inhibitory synapses enables fast removal of chloride from the postsynaptic neuron after GABAAR activation. On the other hand, KCC2 is less densely distributed in the dendritic shafts and serves to slowly regulate chloride throughout the neuron [25]. Organization into nanodomains is influenced by membrane curvature, lipid composition, and cytoskeletal anchorage. These factors create stable platforms for the functioning of KCC2 [26].

Different types of compartmentalizations of KCC2 exist in specialized areas. At the axon initial segment, KCC2 is organized into cytoskeleton anchored lattices that help establish inhibitory control over action potential initiation [27]. In dendritic spines, KCC2 is confined to the geometry of the spine head and increases the magnitude of the chloride gradient. This increases the speed and localization of the regulation of synaptic integration. Dendritic spines are also rich in organelles associated with metabolic and calcium microdomains that modulate the activity of KCC2 [28,29].

Organization of KCC2 also exists at the neuron-glial interface. Perisynaptic astrocyte processes form extracellular ionic boundaries by coordinating transport of water and ions. Hybrid microdomains formed by neuronal transport of chloride and glial buffering of chloride shape local chloride dynamics [30,31]. Finally, organization of KCC2 is dynamic. Changes in activity induce changes in the structure and lipid composition of the neuron. These changes continuously redistribute KCC2, allowing for adaptation of chloride homeostasis to varying microcircuit demands [32].

**Figure 1:**
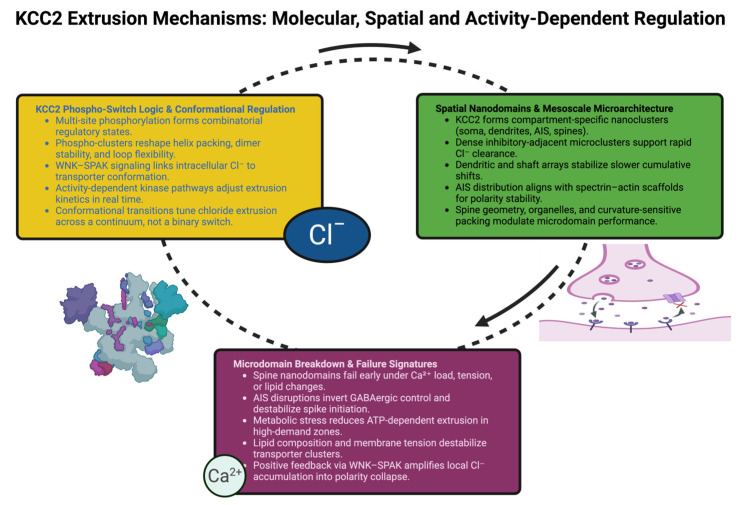
KCC2 extrusion is regulated by: (1) combinatorial phospho-switch states controlling conformation and kinetics; (2) compartment-specific nanodomains stabilizing distinct extrusion regimes; and (3) microdomain failure signatures driven by calcium load, AIS instability, metabolic stress, and lipid remodeling. These layers jointly define the dynamic landscape of neuronal chloride homeostasis.

Figure 1 illustrates how the function of KCC2 is generated by the integration of phospho-switch logic, spatial organization into nanodomains-based extrusion platforms, and micro-domain-specific vulnerabilities, and thus dynamically regulates chloride extrusion.

### 2.3. Microdomain-Specific Breakdown, Failure Signatures, and Hierarchical Collapse Mechanisms

KCC2 dysfunction usually appears as localized failure of microdomains rather than as a general loss of function. These failures occur through a hierarchy of vulnerabilities established by synaptic activity, metabolic demand, cytoskeletal organization, and membrane composition [33]. Dendritic spine nanodomains are particularly sensitive to changes in calcium dynamics and membrane remodeling. Disruption of the extrusion capacity of these domains during high intensity excitatory activity leads to early disruptions in local integration [34,35].

Even mild dysfunction in KCC2 function in the axon initial segment is sufficient to disrupt spike threshold control. Therefore, this compartment can serve as an early indicator of network vulnerability [36]. Additionally, metabolic stress introduces further spatial heterogeneity. Regions with high extrusion demands are dependent upon local ATP production. When there is a reduction in local ATP due to metabolic stress or mitochondrial dysfunction, KCC2 is reduced to low flux states. This generates compartment-specific instability [37]. Additionally, disruptions in lipid composition can destabilize nanodomain formation and decrease extrusion efficiency [38,39].

Feedback mechanisms can further increase dysfunction. Enhanced sensitivity to chloride of the WNK-SPAK pathway may transform small elevations in intracellular chloride into further suppression of KCC2 activity. This creates positive feedback loops that rapidly cause local instability [40].

Therefore, KCC2 dysfunction represents a hierarchical collapse of molecular regulation, spatial organization, and metabolic support. These collapses create zones of chloride instability that interact with NKCC1 activity and glial buffering to determine the net polarity and stability of inhibitory signaling.

## 3. NKCC1: Developmental Program, Pathological Reactivation, and Energetic Burden

### 3.1. Transport Kinetics, Osmotic Design Principles, and the Energetic Logic of Inward Chloride Accumulation

Unlike KCC2, NKCC1 develops an inward Cl− gradient using the energy dependent, coupled cotransport of Na+ and K+, generated from their respective electrochemical gradients. Therefore, the function of NKCC1 is tightly linked to the membrane potential and the metabolic state of the cell through the Na+/K+ ATPase [41]. NKCC1 functions over multiple kinetic ranges depending on osmotic conditions (cell volume), intracellular sodium concentrations, and depolarization of the membrane. For example, hypertonic conditions increase the number of membrane bound NKCC1 units, which increases the rate of Cl-uptake, thus linking the transport of ions to changes in cell volume [42,43].

At the molecular level, NKCC1 couples the binding of Na+, K+, and Cl− within a single binding site; the dynamics of the transport are very sensitive to the concentration of intracellular Na+. This creates a tight coupling between the metabolic state of the cell and the amount of Cl− loaded into the cell. If sufficient ATP is present to support the cotransport, then it occurs efficiently. However, if the metabolic state of the cell becomes stressed, it will alter both the kinetics of the cotransport and the equilibrium for Cl− at the location where NKCC1 is functioning [44,45]. At the microdomain level, NKCC1 combines the metabolic signals generated by the influx of Na+ due to excitatory activity with the electrical signal. Thus, during excitatory activity, increased Na+ influx will enhance the loading of Cl−, allowing NKCC1 to dynamically regulate the inhibitory polarity of the neuron based upon the requirements of the synapse [29,46].

### 3.2. Developmental Blueprint, Chloride Polarity Programs, and Maturation of Inhibitory Computation

Early in development, NKCC1 generates high intracellular Cl− levels, thereby generating depolarizing GABAergic responses that promote the maturation of networks. These excitatory GABAergic responses stimulate Ca++ entry, gene transcription, synaptic formation, dendritic outgrowth, and the migration of interneurons [47]. The transition from an excitatory GABAergic response to a mature inhibitory response is dependent upon the simultaneous down-regulation of NKCC1 and the up-regulation of KCC2 [48].

The timing and spatial heterogeneity of these processes vary among different populations of neurons and between the compartments of individual neurons [49].

Specifically, while the overall trend for the majority of neurons is toward the maturation of inhibitory signaling, some compartments of individual neurons continue to express NKCC1, including growth cones and branch points, and therefore continue to refine structural components locally. Glial cells may continue to express NKCC1 in the adult brain, and participate in regulating ionic composition in the extracellular space, and in modulating the dynamics of the network [43,50].

It is essential to note that this developmental polarity program remains accessible in the adult brain. Under pathological conditions, NKCC1 can be re-expressed in a pattern similar to that observed in the developing brain. This indicates that chloride polarity is not static but continues to be dynamic [51].

### 3.3. Pathological Reactivation, Chloride Loading, and the Emergence of Hyperexcitable Microcircuits

Pathologically, NKCC1 is often up-regulated in a spatially-selective fashion, resulting in localized Cl− loading and polarity reversal. Within dendrites, this results in the conversion of inhibitory input into depolarizing input, and disrupts the ability of neurons to integrate synaptic activity and generate feedback inhibition [52,53].

Astrocytes contribute to this process by up-regulating NKCC1 in response to inflammatory or mechanical injury to the astrocyte, and altering the ionic composition in the extracellular space. Astrocytes may act to disrupt the buffer capacity of neurons to extrude ions, and to disrupt the balance between neuronal ion extrusion and glial ion buffering [54,55].

Metabolic and ionic disturbances further enhance the activity of NKCC1. Sustained Na+ influx enhances Cl− loading, especially when KCC2 activity is compromised. This results in rapid fluctuations in chloride polarity, promoting hypersynchronous activity and pathological oscillations [56,57]. The purpose of the next Table 1 is to highlight how NKCC1 integrates ion cotransport physics, developmental polarity programs, and pathological re-activation to regulate chloride homeostasis in both neuronal and glial compartments.

Therefore, the re-expression of NKCC1 underlies many aspects of the pathology associated with altered metabolic states and structural plasticity. By recreating the chloride loading states of early developmental stages, NKCC1 contributes to the disruption of inhibitory precision, and in conjunction with the disruption of KCC2 function, facilitates the propagation of local disturbances into widespread dysfunction of the circuit [68].

## 4. Astrocytic Chloride Microdomains and the Tripartite Regulation of GABAergic Microinhibition

### 4.1. Astrocytic Chloride Transport Machinery and the Architecture of Perisynaptic Ionic Microdomains

Microdomains in the astrocyte plasma membrane provide a site for the integration of ionic, metabolic and structural signals in response to synaptic activity. The primary method of regulating ionic concentrations and maintaining the local ionic environment surrounding inhibitory synapses involves the regulation of chloride (Cl−) flux by astrocytes. Astrocytes utilize several routes to modulate chloride concentrations in the extracellular space; namely, volume-regulated anion channels (VRACs), calcium-activated chloride channels (CaCCs), ligand-gated chloride channels (LCCCs) and electroneutrally-cotransported ions. Each of these chloride-regulatory pathways has the capacity to modulate the extracellular Cl− concentration in a distinct way [69]. Recent studies using high-resolution imaging techniques have shown that these pathways are not randomly distributed throughout the plasma membrane of astrocytes but instead are organized into nano-domains that are located proximal to inhibitory synapses [70,71].

Each domain exists within a limited area of the extracellular space with a defined geometry that restricts diffusion. Therefore, the regulation of Cl− flux by astrocytes can be modulated indirectly through the regulation of the relative concentrations of co-transported ions such as potassium (K+) and sodium (Na+), and bicarbonate (HCO−3). This regulation of the extracellular ionic environment is important for the regulation of GABA(A) receptor-mediated responses, particularly because the amplitude and kinetics of GABA(A)-mediated responses depend on the extracellular concentration of Cl− [72].

Regulation of the extracellular Cl− concentration by astrocytes occurs through the modulation of chloride channel activity. Chloride channel activity is regulated in part through the mechanical properties of the plasma membrane of astrocytes [73]. Cholesterol-rich microdomains within the plasma membrane of perisynaptic astrocytic processes contain aquaporin and other ion-transporting proteins and thus serve to couple chloride flux to water flux. Such microdomains provide dynamic “tunnels” through which the activity of inhibitory synapses rapidly and reversibly generates Cl− accumulation in the extracellular space [10].

Because perisynaptic astrocytic processes extend extensively throughout the synaptic neuropil, these microdomains provide a mechanism by which astrocytes can modulate the kinetic characteristics of GABAergic transmission and the relative magnitudes of depolarizing and hyperpolarizing responses produced by GABA(A) receptors [74]. In addition, the organization of these microdomains appears to be responsive to mechanical force, glial calcium wave activity and metabolic status. Thus, the regulation of Cl− by astrocytes is dynamically controlled in accordance with changing network conditions [75].

### 4.2. Glial–Neuronal Coupling, Microdomain Compartmentalization, and Modulation of Inhibitory Precision

The morphological characteristics of astrocytes provide a spatial framework that enables the regulation of Cl− by astrocytes to be integrated into a larger system of compartmentalized microdomains. Perisynaptic astrocytic processes produce geometrically-complex three-dimensional environments that constrain the movement of Cl− and other co-transported anions (e.g., bicarbonate) away from GABAA receptors following GABAergic conductance activation. Depending on the particular geometry of the extracellular space, the movement of Cl− and other ions can either be facilitated or restricted by the geometric constraints imposed by the perisynaptic processes [76]. By regulating the distribution of Cl− within such microdomains, astrocytes are able to regulate the amount of Cl− that reaches GABAA receptors, and thus can modulate the efficacy of GABAergic transmission with great precision. Additionally, local increases in intracellular calcium (Ca2+) can induce small-scale changes in the shape of astrocytes that can also modulate the degree of tortuosity of diffusive paths and the extracellular volume in the vicinity of inhibitory synapses. Both factors can regulate the lifetime and amplitude of Cl− transients associated with inhibitory synapses [77].

Astrocytes also play a significant role in modulating presynaptic aspects of GABAergic function. Many inhibitory axon terminals possess presynaptic receptors that are sensitive to Cl− and whose activation is contingent on the presence of Cl− in the perisynaptic space. Small-scale adjustments in extracellular Cl− levels produced by astrocytes can thus modulate the resting membrane potential of boutons, and subsequently, the probability of GABA release [78]. Such a regulatory loop represents a bi-directional relationship wherein the activity of inhibitory inputs modulates the ionic state of astrocytes, and the ionic state of astrocytes, in turn, modulates the subsequent inhibitory output. Manipulations of proteins involved in chloride regulation in astrocytes have provided evidence that the variability in GABA release at individual synapses can be influenced by ionic microdomain interactions, and therefore demonstrate a previously under-appreciated level of glial influence on the precision of inhibitory transmission [79].

In addition to directly regulating Cl− flux, astrocytes also regulate bicarbonate-dependent mechanisms that modulate the polarity of GABAergic transmission. Carbonic anhydrase enzymes that are present in the fine astrocytic processes surrounding synapses regulate the local bicarbonate concentration by generating or consuming bicarbonate, and thereby modulate the ionic composition of the extracellular fluid available for GABA(A) receptor-mediated conductance [80]. Since bicarbonate and Cl− have differing effects on the magnitude and duration of inhibitory currents, astrocytic regulation of bicarbonate availability can also modulate the efficacy of inhibitory transmission in a spatially-restricted, yet physiologically-relevant, fashion. Collectively, these data suggest that astrocytes regulate inhibitory synapses by coordinating the regulation of Cl−, bicarbonate and the extracellular geometry surrounding them, to form a three-part structure in which glial microdomains function as ionic integrators [81].

### 4.3. Microdomain-Specific Astrocytic Failure and Its Propagation Through Inhibitory Networks

Early indicators of dysfunction of the regulatory mechanisms employed by astrocytes to regulate chloride often occur at specific microdomains in which structural alterations in the cytoskeleton result in the loss of stability of perisynaptic processes. Such loss of stability causes a failure of the spatial confinement necessary to generate precise chloride microgradients in the extracellular space. Consequently, Cl− accumulates persistently in the extracellular space at levels greater than those predicted by GABAergic conductance [82]. Ultimately, such structural changes result in disruptions to the ionic coupling between glial and neuronal compartments and disrupt the regulatory mechanisms employed by astrocytes to maintain inhibitory synaptic reliability [83].

Persistent inflammatory signals introduce additional vulnerabilities to the functioning of the astrocytic chloride regulatory machinery. Activation of inflammatory signals can modulate the functioning of astrocytic chloride channels and cotransporters. For example, activating VRACs in pathological conditions can release large quantities of Cl− into the extracellular space and, thereby, alter the local ionic landscape in a manner that compromises both inhibitory and excitatory balance [84]. Conversely, reactive astrocytes can increase expression of NKCC1, resulting in increased Cl− in compartments that normally maintain low concentrations of Cl−. Such a reorganization of the chloride gradient between astrocytes and neurons can impact how extracellular Cl− equilibrates following inhibitory bursts, creating microdomains in which inhibitory input exhibits abnormal or contradictory properties [85].

Metabolic stress is another condition in which the functioning of astrocytes in regulating chloride can be compromised. Under normal conditions, astrocytes support inhibitory circuits by providing energy substrates, buffering extracellular ions, and regulating extracellular water balance. However, when energy availability is constrained, the functioning of astrocytes in regulating chloride is severely impaired. Therefore, astrocytes are unable to regulate the extracellular composition during episodes of high-frequency interneuron firing. If prolonged periods of reduced energy availability are maintained, it can result in incomplete removal of perisynaptic Cl− transients, and ultimately result in gradual changes in inhibitory polarity in adjacent neuronal microdomains. While the initial changes may remain localized, they can eventually propagate through microcircuits as adjacent synapses are exposed to altered ionic conditions, thereby compromising both feed-forward and feedback inhibition [86].

Collectively, the cumulative effects of microdomain-specific failures of astrocytes are that they lose the ability to buffer fluctuations in chloride associated with inhibitory computation. Instead of decreasing the variability in the ionic composition, dysfunctional astrocytic processes allow for the creation of “chloride hotspots” that perturb the timing and stability of GABAergic signaling. As these perturbations accumulate along dendritic trees and axonal zones, they can disrupt the synchronization and gain-control mechanisms that rely on stable inhibitory conductance. Furthermore, when these local imbalances in astrocytic chloride regulation are combined with vulnerabilities in transporter function in neurons, they can further destabilize the entire network [87,88].

**Figure 2:**
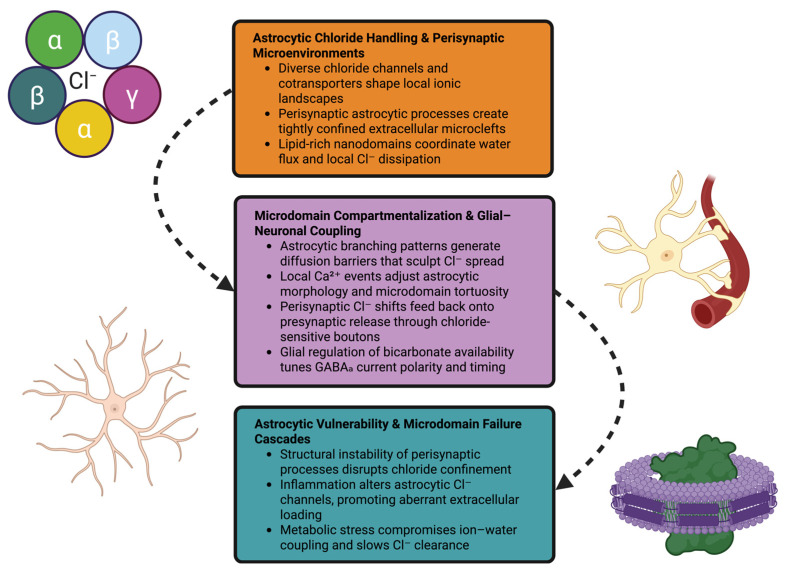
A tripartite schematic illustrating: (1) astrocytic chloride-handling machinery and formation of perisynaptic ionic niches; (2) microdomain compartmentalization that shapes chloride spread and modulates GABAergic precision; and (3) failure cascades where structural, inflammatory, or metabolic stress disrupts chloride buffering, enabling instability to propagate through inhibitory networks.

Astrocytes create a unique ionic environment surrounding inhibitory synapses through the use of multiple types of chloride channels, cotransporters, and lipid-based microdomains. These perisynaptic structures regulate chloride flux, modulate the spread of GABA-induced signals, and couple glial and neuronal physiology at the micrometer scale. Structural, metabolic, or inflammatory disruption of these microdomains produces localized failures of the functioning of these microdomains, which are transmitted through the network and contribute to broader instability in inhibitory transmission (Figure 2).

These data collectively indicate that chloride-containing microdomains in astrocytes function as critical stabilizing components of inhibitory networks, mediating the interaction of ionic, metabolic and structural signals along synaptic, metabolic and structural axes. When these glial functions are compromised, microdomain-specific deficiencies in chloride regulation can be propagated through microcircuits in ways that greatly enhance the propensity of the network to hyperexcitability [89]. The next section will discuss how astrocytic chloride regulation influences neuronal chloride transport to assess the implications of deficiencies in chloride regulation in inhibitory microdomains for network-level instability and circuit collapse.

## 5. Microcircuit Consequences: How Aberrant E_Cl_ Dynamics Collapse Cortical Computation

### 5.1. Compartment-Specific Degradation of Inhibitory Precision and Emergence of Chloride-Driven Polarity Mosaics

In comparison to the traditional understanding of dendritic computation as being based upon the spatial distribution of electrical signals (i.e., the structural properties of dendrites), in cases of localized variations in ECl, dendritic computations occur in asynchronous, micro-domain-based sub-spaces. Micro-domains are characterized by their unique ion composition rather than the structural characteristics of the dendritic tree. For example, the same excitatory stimulus could generate a strong shunt response in one micro-domain but a weak hyperpolarizing current in another [90]. Because the strength of GABAergic currents depends on ECl, changes in ECl of only a few millivolts can convert GABAergic responses from hyperpolarizing to depolarizing or shunting. This can result in distorted local integration at sites of excitatory convergence. Similar distortions of function will occur in AISs, where inhibition exerts tight control over the initiation of spikes. For instance, small changes in ECl can reverse the impact of axo-axonal inhibition on spike generation from facilitory to suppressive [28].

Additionally, changes in ECl can disrupt spike timing, and therefore the capacity of a neuron to participate in circuits that operate at gamma frequencies. Therefore, in addition to disrupting spike timing, changes in ECL can also disrupt the capacity of a neuron to maintain the temporal integrity of feed forward inhibitory motifs and allow timing errors to be rapidly propagated through local circuits [26,91].

In addition to influencing the postsynaptic membranes, chloride also has an effect on the presynaptic terminals responsible for the release of neurotransmitter from the inhibitory boutons. Inhibitory boutons contain chloride-sensitive mechanisms that regulate the membrane potential and calcium-dependent release probabilities of inhibitory transmitters. Therefore, GABAergic output is directly related to the local ionic environment. The presence of astrocytes in the vicinity of pyramidal cells and the glial accumulation of chloride via the NKCC1 transporter provide an additional source of variability in the local ionic environment that can further dissociate the temporal coordination of interneuron-pyramidal cell interactions [92].

### 5.2. State-Dependent Vulnerability: Dendritic Gating Distortions, Oscillatory Fragmentation, and Compromised Temporal Motifs

Abnormalities in chloride levels are more detrimental when rapid inhibitory calibration is required. In other words, when the neuron is in an active integration mode, excitatory inputs produce plateau potentials that are typically regulated by inhibitory influences. When chloride accumulates, inhibitory efficacy decreases and plateau potentials persist longer than usual. Consequently, the time-voltage integrals that determine synaptic plasticity and spike output are modified [93].

Similar to how oscillations depend on the same factors as excitability and spike frequency adaptation, oscillations depend on the timing of inhibitory currents as well as the relatively consistent amplitude of these currents during the cycle for maintaining oscillatory activity. However, disturbances in chloride gradients alter the decay rates of inhibitory currents, distort the phase of oscillatory activity, and change the ratio of chloride and bicarbonate conductance through GABAA receptors, resulting in fragmentation of oscillatory activity and decreased synchronization [14,94].

Quiescent to active processing represents the highest risk of disturbances in chloride levels. That is, the rapid increases in the rate of firing of interneurons cause temporary increases in the chloride loads that must be quickly removed. If the removal of chloride does not balance the buffering provided by astrocytes, temporary imbalances in ECl will develop, and the inhibitory control required to stabilize activity will be disrupted. The disturbances in inhibitory control will lead to poor signal discrimination, reduced gain control, and poor pattern separation [95,96].

Disturbances in chloride levels pose significant danger for circuits that require millisecond resolution such as coincidence detection and feed-forward pathways. Disturbances in chloride levels can produce subtle changes in the timing of neuronal responses that can modify the operating regimes of the neurons participating in coincidence detection and feed-forward pathways, reduce synchronization among population responses, and disrupt the maintenance of assemblies of neurons supporting attention and working memory [97,98].

### 5.3. Propagation to Network-Level Failure: Recurrent Amplification, Attractor Destabilization, and Collapse of Cortical Stability

Although localized, disturbances in chloride levels will propagate through the recurrent networks of the cortex. Polarization distortions caused by disturbances in chloride levels will enhance the excitatory drive of downstream neurons and alter the recruitment thresholds of interneurons, especially for inhibitory populations that are energetically expensive and whose ECl values are variable and therefore amplify disturbances across the cortical territories [99].

At the systems level, the instability caused by disturbances in chloride levels will decrease the stability and resistance to premature transition of the attractor dynamics that sustain activity and controlled state transitions. Introducing noise in the attractor landscape by variability in chloride concentration will reduce the stability of the attractor and increase its susceptibility to premature transition. Therefore, the stability of working memory will be compromised, the robustness of sensory representations will be compromised, and the temporal organization of network trajectories will be compromised [100].

Additionally, the large-scale coordinated oscillation will be disrupted. The disruption of synchrony of interneurons will break the coherent gamma activity into desynchronized local domains, decrease the theta-gamma coupling, and disrupt the beta-mediated long-distance communication. Therefore, the hierarchical temporal organization of the information transmission in the cortex will be compromised in terms of both bandwidth and reliability [101].

Table 2 illustrates the multi-scale failure modes that connect the dysregulation of chloride microdomains to the breakdown of cortical computational processes.

Therefore, the multi-scale disturbances described above will converge to form a critical regime of cortical instability. Cortical networks will be made hypersensitive to disturbances, the reliability of inhibitory systems will be compromised, and the large-scale coordination will be disrupted. Furthermore, in this regime, small disturbances in chloride microdomains will produce large functional consequences. Therefore, the multi-scale disturbances of chloride microdomains represent a common mechanistic basis for the clinical manifestations of various disorders, including seizure susceptibility, sensory distortion, and cognitive fragmentation, which arise from disturbances of chloride homeostasis [116,117].

## 6. Disease Convergence: Chloride Microdomain Dysregulation Across Human Pathologies

### 6.1. Epilepsy, Neonatal Seizures, and Focal Cortical Dysplasias: Microdomain Polarity Shifts as a Unifying Ictogenic Substrate

Epilepsy, regardless of type, occurs by the exact same mechanisms. This mechanism of seizure generation is based on the convergence of local alterations in chloride, causing local increases in ECl and polarity reversals in both dendritic and perisomatic microdomains. All types of epilepsy modify KCC2 differently. However, all types of epilepsy may cause instability of KCC2, whether through modifying KCC2 posttranslationally, phosphorylating KCC2 to inhibit it, or through interference with the metabolic process [118,119]. When local chloride efflux capabilities cannot counteract chloride accumulation, even small amounts of accumulated chloride will cause depolarization of spike timing and diminish the precision of inhibitory circuitry. In addition to the limited diffusion of chloride and the limited buffering capacity of astrocytes for chloride, the retention of compartmental chloride will further promote the creation of branch-specific loss of gain control in parvalbumin interneurons. The retention of compartmental chloride, due to the conditions that create compartmental chloride, will also create the “pre-ictal corridors” of vulnerable microdomains that will facilitate seizure spread based on energy [120].

Due to the relatively immature state of the neuron in the developing brain, the developing brain has a greater potential for producing seizures due to the high levels of NKCC1. The heterogeneous accumulation of chloride in the proximal dendrite and the growth-related regions of the immature neuron are influenced by local ionic gradients that favor chloride uptake [121,122]. Regions of the neuron where chloride is accumulated to a greater extent will become “hot-spots” and will convert GABAergic signaling into depolarizing and regenerative signals. Network synchrony, produced by the localized signals, will help to facilitate ictal recruitment. Continued expression of the developmentally abundant NKCC1 domain beyond its normal developmental time point will increase the possibility for abnormal excitatory coordination and increase the chance of seizures [36].

Focal Cortical Dysplasias are created by the alteration of regulation of chloride. The combination of the ectopically-expressed NKCC1 and the altered KCC2 nanodomain architecture, combined with the reduced and simplified astrocyte structure and reduced diffusion rates of the extracellular space will increase the potential for prolonged chloride retention and stabilize the depolarized microdomains. The combination of the two neuro-glial phenotypes will produce chronic distortions of polarity that will produce chronic epileptic activity [123,124].

### 6.2. Neuropathic Pain: Chloride-Dependent Disinhibition as a Cortical Gain-Amplifying Mechanism

Depolarizing GABAergic signaling acts as a gain-amplifying mechanism in neuropathic pain. The decrease in KCC2 by microglia in dorsal horn neurons will result in depolarizing GABAergic responses and stimulate nociceptive transmission. Decreasing the number of KCC2 enriched dendritic nanodomains in pyramidal neurons will produce localized chloride accumulation, prolong depolarization, and increase the synchronization of nociceptive assemblies [125].

Additionally, reactive astrocytes will contribute to this process by increasing NKCC1 and by removing perisynaptic processes. Removing perisynaptic processes will remove the capacity of the astrocytes to buffer the extracellular space. The subsequent accumulation of localized chloride transients will decrease the sharpness of the inhibitory circuits and increase the area of the activated region of cortex. Thus, in this case, chloride dysregulation functions as a continued gain-amplifier that will allow the aberrant representation of sensory information and sustain the chronic condition of pain [126,127].

### 6.3. Schizophrenia-Spectrum Conditions: Chloride Microdomain Instability as a Determinant of Cortical Dysconnectivity

Instability of chloride microdomains will disrupt the function of interneurons and lead to large-scale network disruption in individuals with schizophrenia spectrum disorders. Reductions in KCC2 expression in parvalbumin interneurons and the focal enrichment of NKCC1 in dendrites of pyramidal cells will create heterogenously-polarized states that will hinder inhibitory timing. Hindering inhibitory timing will directly relate to the dependence of gamma oscillations on tightly-regulated ECl. A slight depolarizing shift in ECl will disrupt the timing of the oscillations and decrease the network coherence needed for working memory and predictive processing [128,129].

Localized NKCC1-enriched dendritic segments of pyramidal cells will hinder inhibitory integration and destabilize repetitive patterns of network activity. The increased propensity of the network to random fluctuations in synaptic activity and the decreased ability to sustain activity are indicative of cortical dysconnectivity [130,131].

## 7. Therapeutic Frontiers: Rebuilding Chloride Stability Through Precision Microdomain Engineering

### 7.1. Reconstituting KCC2 Function Through Phospho-Logic Repair, Structural Stabilization, and Microdomain-Specific Reactivation

Therapies for KCC2 are transitioning from the regulation of KCC2’s expression levels to repair the regulatory architecture of KCC2. High resolution phospho-mapping has revealed several allosteric networks that regulate the states of KCC2. New small molecule agents use these allosteric networks to maintain the optimal conformational states of KCC2 to maximize the functional output of Cl−/HCO−3 − exchange without changing the total amount of KCC2 membrane-bound [30,132,133].

Advances in the regulation of the WNK-SPAK pathway have illustrated the importance of spatial specificity. Rather than generally inhibiting kinases, current therapies selectively inhibit kinases within specific subcellular compartments. Kinase activity is preserved within other compartments; however, the inhibitory constraints of KCC2 are removed from the inhibited kinases [134,135].

Dephosphorylation of KCC2 in KCC2-rich domains can be achieved by using phosphatases to reactivate localized KCC2 [136].

A second therapeutic approach to address the structural stabilization of KCC2 nanodomains. Super-resolution microscopy has demonstrated that membrane curvature, lipid composition and cytoskeletal scaffolding are key components of the structural requirements for KCC2 clustering. Agents that reassemble the nano-architecture of KCC2 nanodomains, including lipid-modifying drugs, scaffold-stabilizing peptides and oligomer-enhancers, will enable compartment-specific chloride efflux and preserve the precision of inhibitory microcircuits [137,138,139].

### 7.2. Modulating NKCC1-Driven Chloride Loading Through Developmental Recalibration, Metabolic Engineering, and Glial-Selective Interventions

The therapeutic approaches to modulate NKCC1 are transitioning toward selective and context-dependent methods. Agents that change the binding properties of NKCC1 or the ion-binding geometry of NKCC1 will reduce chloride accumulation while maintaining the core function of NKCC1 in cellular homeostasis. The separation of NKCC1 pools in neurons and glial cells and the different subcellular compartments of NKCC1 will be achieved by targeting different isoforms of NKCC1 [140,141].

Recalibrating the developmental timing of the down-regulation of NKCC1 represents a developmental recalibration strategy. Once the mature chloride states continue beyond their normal developmental times, epigenetic modifiers can reset the developmental timing to reduce depolarizing GABAergic signaling while permitting normal growth processes [142,143,144].

Modifications of metabolism represent another regulatory layer. Since NKCC1 activity is dependent on the gradients generated by the Na+/K+ ATPase, improving the local availability of ATP, such as through targeted placement of mitochondria, will impact chloride loading at the microdomain level [145]. Reactive astrocytes can be targeted with glial-selective strategies to restore the extracellular ionic balance by limiting excessive NKCC1 activity and restoring the capacity of astrocytes to buffer the extracellular space [9,43].

### 7.3. Engineering the Chloride Milieu Through Astrocytic Remodeling, Extracellular Geometry Tuning, and Synthetic Ionic Support Systems

There are numerous innovative and creative therapeutic concepts under development that are more than simply the modification of transporters. These are the design of the spatial organization of ionic homeostasis. Strategies of astrocytic remodeling will seek to restore the complex architecture of astrocytes surrounding inhibitory synapses and restore diffusion pathways. Modifying the cytoskeleton, lipid rebalancing and regulating aquaporins will enable improved rates of chloride dissipation and better fidelity of spatiotemporal inhibitory signaling [146].

Geometry tuning of the extracellular space is an additional layer of control over the dynamics of chloride accumulation. Geometry tuning of the extracellular space provides a means of controlling the volume, tortuosity and water flux of the extracellular space and controlling the dynamics of chloride accumulation and preserve inhibitory polarity during heightened periods of network activity [147].

Synthetic Ionic Support Systems (SISS) are a new category of engineered solutions to problems of microdomain stability. SISS include nanoparticles engineered to buffer chloride in localized areas; conductive peptide assemblies capable of modulating chloride flow; and responsive ion channels capable of dynamically modulating microdomain stability. Gene editing technologies that target KCC2 variants or aberrantly expressed NKCC1 are long term strategies to re-engineer the molecular architecture of chloride microdomains [148,149].

## 8. Conclusions

The view that chloride homeostasis is a relatively stable physiological background variable has been challenged by new evidence that demonstrates that chloride’s spatial organization and regulation are critical to its role in defining and regulating inhibitory signals. Thus, we propose that chloride is not simply an inhibitor of excitability, but rather is involved in shaping the microenvironmental conditions under which inhibitory signals succeed or fail.

Chloride’s microenvironment is shaped by three main factors: the spatial distribution of transporters, the microanatomical architecture of neurons and glia, and the geometry of glial cells (astrocytes). These factors create a complex array of microdomains in which chloride behaves differently. In each domain, chloride creates dynamic gradients that are dependent on ongoing synaptic activity. The loss of structural coherence of transporters, or weakening of astrocyte buffering capabilities, can lead to a loss of stability in chloride microdomains, resulting in disease states characterized by abnormal excitability. Disease-induced instability in chloride microdomains can spread from one neuron to another based on their anatomical connections and glial coverage, indicating that chloride plays a significant role in determining the spatial organization of neural circuits’ resilience.

In addition to demonstrating how chloride’s microdomains contribute to disease states such as epilepsy, neuropathic pain and schizophrenia spectrum disorders, we demonstrate that there are multiple forms of human pathology that all appear to involve abnormalities in chloride microdomains. All of these pathologies are associated with disrupted inhibitory precision, disruption of oscillations and disruption of dendritic integration. While the specific characteristics of the disease states vary, they all involve similar disruptions in chloride microdomains. Specifically, all of these pathologies involve a disruption in chloride’s ability to be removed from the cell, increased accumulation of chloride within the cell, decreased buffering capacity of chloride, and/or unstable inhibitory timing.

Given the similarities in the disruption of chloride microdomains in all of these disease states, we hypothesize that chloride may play a unifying physiological role in all of these diseases. The development of novel therapeutic approaches to restore normal function to chloride microdomains in all of these diseases represents a significant opportunity to develop new treatments for a range of neurological diseases that have historically been viewed as unrelated. Examples of these novel therapies include efforts to restore the phosphorylation state of transporters, stabilize KCC2 nanodomains, adjust the developmental trajectory of NKCC1, provide metabolic support, and modify astrocyte microarchitecture to restore chloride homeostasis. We present these examples of therapy as illustrative of a larger movement toward developing therapies that target the microdomain organization of chloride homeostasis, as opposed to simply attempting to restore balance to chloride concentrations. Rather than focusing solely on restoring chloride concentration, our intention is to restore the ionic architecture of microdomains that are required for normal functioning of neural circuits.

While much remains to be learned, the field of chloride biology is currently at a point of significant conceptual growth. Recent advances in imaging technology, organoid models and computational modeling have provided the tools necessary to examine chloride dynamics at a level of detail that was previously impossible. Our intent is to encourage further research into chloride biology that builds upon this framework, recognizing chloride as a dynamic, spatially organized biological variable that is essential for the proper functioning of neural circuits.

If our conclusions seem premature, it may be due to the fact that much remains to be learned about the organization and regulation of chloride microdomains. Chloride microdomains continue to reveal layers of organization that challenge long-standing assumptions regarding the mechanisms of inhibition and network stability. We intend this review to serve as both a foundation and invitation—an encouragement to explore the rapidly evolving field of ionic physiology that will continue to guide the understanding of neural function and dysfunction.

## Figures and Tables

**Table 1:** Core Regulatory Axes of NKCC1 and Their Impact on Chloride Polarity and Network Function. Each entry captures a mechanistic layer through which NKCC1 remodels chloride equilibrium and network dynamics.

Axis	Mechanism	Effect on Cl^−^ Polarity	Systems	References
Transport–Energetics	Na^+^–K^+^–Cl^−^ cotransport; ATP-dependent gradients	Inward Cl^−^ loading; ECl depolarization	NKCC1; Na^+^/K^+^ ATPase	[58]
Osmotic Coupling	Volume-sensitive activation; membrane recruitment	Rapid Cl^−^ influx under stress	Osmosensors; cytoskeleton	[59]
Metabolic Control	ATP availability; mitochondrial support	Energy-dependent inhibitory weakening	Mitochondria; ATP signaling	[60,61]
Developmental Program	Early NKCC1 dominance; regulated decline	Depolarizing GABA → inhibitory maturation	NKCC1 regulators; KCC2	[23]
Spatial Gradients	Compartment-specific expression	Local ECl heterogeneity	Dendrites; axons; glia	[62]
Pathological Reactivation	Injury/inflammation-induced upregulation	GABA polarity inversion	Reactive astrocytes; cytokines	[63]
Neuron–Glia Interface	Astrocytic NKCC1; extracellular modulation	Impaired buffering; shared instability	Astrocytes; perisynaptic space	[64]
Microcircuit Instability	NKCC1↑ + KCC2↓	Hyperexcitability; oscillatory disruption	NKCC1 clusters; KCC2 loss	[65]
Energetic Cost	High ATP demand	Reduced inhibitory resilience	Pump–mitochondria coupling	[66,67]

**Table 2:** Each domain captures a mechanistic step in the breakdown of inhibitory function: dendritic polarity mosaics distort integration, AIS instability disrupts spike timing, presynaptic chloride fluctuations introduce release variability, and state transitions expose latent vulnerabilities.

Failure Domain	Core Ionic/Microstructural Mechanisms	Immediate Microcircuit Consequences	Systems-Level Computational Breakdown	Representative Molecular/Cellular Actors	References
Dendritic Polarity Fragmentation	Local Cl^−^ pooling in thin dendrites; electro-diffusion bottlenecks; NKCC1-driven microdomains; impaired KCC2 extrusion	Mixed shunting–depolarizing inhibition; spurious dendritic spike facilitation; loss of integration coherence	Distorted input weighting; degraded feature tuning; impaired synaptic integration fidelity	Dendritic NKCC1 clusters; KCC2 nanodomains; GABA(A) receptor microdistributions	[6]
AIS Reversal-Point Instability	Millivolt-scale E_Cl drift at AIS; axo-axonic GABA polarity inversion; Na^+^/Cl^−^ microdomain coupling	Lowered spike threshold; unreliable AP initiation; degraded phase-locking and timing precision	Collapse of gamma pacing; weakened feedforward inhibition; reduced temporal resolution of computation	Axon initial segment GABA(A) receptors; ankyrin-G scaffold; Na^+^ channels	[102]
Presynaptic Chloride Vulnerability	Cl^−^-sensitive bouton excitability; gliogenic Cl^−^ fluctuations; NKCC1-dependent terminal depolarization	Variability in GABA release probability; irregular inhibitory drive; altered short-term plasticity	Temporal jitter in inhibition; compromised recurrent stability; breakdown of gain control	Presynaptic Cl^−^ channels; astrocytic NKCC1; perisynaptic glial sheaths	[103,104]
State-Transition Fragility	Rapid Cl^−^ accumulation during high activity; delayed extrusion; astrocytic clearance mismatch	Failure to match inhibition to computational demand; plateau prolongation; burst-prone dendritic states	Loss of pattern separation; degraded attentional gating; sensory over-integration or under-segmentation	Activity-driven Na^+^ influx sites; astrocytic Cl^−^ transporters; Na^+^/K^+^ ATPase	[105,106,107,108,109]
Oscillatory Fragmentation	E_Cl shifts shortening inhibitory decay; altered GABA(A) Cl^−^ vs. HCO_3_^−^ ratio; interneuron-specific Cl^−^ instability	Phase jitter in gamma; weakened theta pacing; unstable beta synchrony	Breakdown of coherence-based communication; impaired rhythmic coordination; disrupted hierarchical coupling	PV interneurons; SST interneurons; bicarbonate conductance networks	[110]
Coincidence & Timing Disruption	Sub-millisecond polarity shifts across dendritic branches; local Cl^−^ mosaics; Na^+^-linked facilitation of depolarizing inhibition	Impaired coincidence detection; timing drift; inconsistent firing-mode transitions	Degraded population precision; reduced spike-time reliability; impaired working-memory stability	Dendritic spike zones; feedforward inhibitory microcircuits	[111]
Recurrent Loop Destabilization	Excitability escalation in recurrent excitatory loops; interneuron Cl^−^ overload; compromised inhibitory recursion	Positive-feedback amplification; runaway dendritic depolarization; persistent hyperexcitability	Collapse of attractor depth; noisy state transitions; instability of persistent activity	Recurrent pyramidal networks; interneuron hubs; NKCC1-reactivated domains	[112]
Attractor & Representational Collapse	Polarity noise perturbing integration windows; microdomain Cl^−^ volatility; variable inhibitory gain	Shallow attractor basins; premature escape from memory states; unstable sensory maps	Working-memory decay; impaired predictive coding; perceptual unreliability	Layer-specific inhibitory motifs; long-range integrative circuits	[113,114]
Large-Scale Synchrony Failure	Phase misalignment across modules; variable interneuron excitability; heterogeneous E_Cl drifting	Fragmented cortical rhythm fields; desynchronized assemblies; reduced information bandwidth	Network-wide instability; impaired global integration; increased susceptibility to noise & seizures	Long-range GABAergic projections; thalamocortical loops; myelinated inhibitory axons	[115]
Systemic Cortical Collapse	Widespread chloride-driven depolarizing inhibition; KCC2/NKCC1 imbalance; metabolic load	Near-critical instability; hypersensitivity to small perturbations; impaired signal–noise segregation	Seizure vulnerability; cognitive fragmentation; breakdown of stable computation	Multicompartment neurons; astrocyte–neuron ion coupling; chloride-transport machinery	[16]

## Data Availability

No new data were created or analyzed in this study. Data sharing is not applicable to this article.
